# Medicare Part D Savings Under the Manufacturer Discount Program vs Coverage Gap Discounts

**DOI:** 10.1001/jamanetworkopen.2025.30778

**Published:** 2025-09-08

**Authors:** Benjamin N. Rome, Katherine R. Garrett, Luca Maini

**Affiliations:** 1 Program on Regulation, Therapeutics, And Law (PORTAL), Division of Pharmacoepidemiology and Pharmacoeconomics, Department of Medicine, Brigham and Women’s Hospital and Harvard Medical School, Boston, Massachusetts; 2 Department of Health Care Policy, Harvard Medical School, Boston, Massachusetts

## Abstract

This cross-sectional study estimates spending changes for the Medicare Part D program after the Manufacturer Discount Program replaces the Coverage Gap Discount program.

## Introduction

Starting in 2025, the Manufacturer Discount Program (MDP) required brand-name prescription drug manufacturers to offer discounts for their drugs to be covered in the Medicare Part D program: 10% of costs once patients reach the deductible ($590 in 2025) and 20% once patients exceed an annual out-of-pocket maximum ($2000 in 2025). This new program, enacted under the Inflation Reduction Act of 2022, replaced the Coverage Gap Discount (CGD) program, which had required branded drug manufacturers to subsidize up to 70% of costs for claims in the coverage gap. The shift from the CGD to the MDP might have important consequences for Medicare spending on prescription drugs and for which drugs trigger the greatest discounts.

## Methods

This cross-sectional study used a 20% sample of 2022 Medicare Part D prescription drug claims to compare actual CGD payments with estimated payments had the MDP been in place that year instead. We included covered drugs that incurred CGDs from 2012 to 2022; the eligibility is similar for the 2 programs.^1^ Estimated MPD payments were simulated as 10% of costs after patients reached the deductible ($536) and 20% after reaching the out-of-pocket maximum ($1817); both thresholds were adjusted from 2025 to 2022 US dollars using the Consumer Price Index.^2^ Per federal guidance, out-of-pocket costs contributing to the threshold included 100% of costs during the deductible plus 25% after the deductible, even if patients actually paid less based on their plan’s design.

To understand which types of drugs were most affected by the change, we stratified results in quartiles by the number of Medicare beneficiaries using the drug, Medicare spending (before rebates), and mean spending per patient (ie, price). Data were aggregated at the drug level using data from SSR Health.^3^ Data analysis was performed from November 2024 to March 2025 using Stata version 18 (StataCorp). The study follows STROBE reporting guidelines; the use of Medicare claims data was approved as exempt research by the Harvard Medical School institutional review board.

## Results

Of $244.4 billion in prerebate Part D spending in 2022, $205.5 billion (84%) was spent on brand-name drugs that owed CGDs. Manufacturer CGD discounts totaled $16.8 billion (8% of gross spending), and estimated MDP discounts would have totaled $34.5 billion (17% of gross spending).

Discounts under the MDP were higher than under the CGD program for 802 of 902 drugs (89%). Drugs with the highest CGDs, including top-selling anticoagulants and diabetes medications, owed similar discounts under both programs (Figure, A). By contrast, drugs with prices greater than the median ($5526/y) owed relatively small CGDs but would have owed approximately 20% under the MDP (Figure, B).

**Figure.**
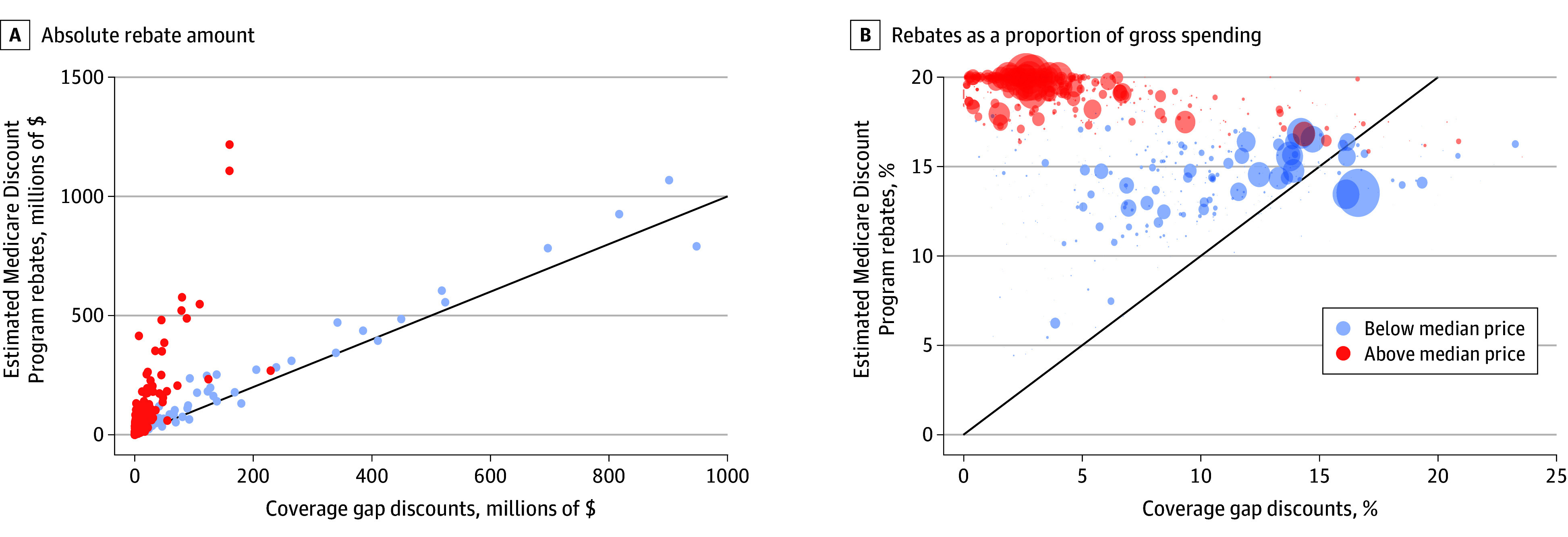
Comparison of Coverage Gap Discount vs Medicare Discount Program Rebates Each dot or bubble is a single brand-name drug, defined based on SSR Health^3^; bubble sizes in panel B reflect gross spending in 2022. Red dots and bubbles represent drugs with mean spending per beneficiary (price) higher than the median of $5526 per year. Panel A excludes 1 outlier product (apixaban), which paid $2.5 billion in Coverage Gap Discounts in 2022 and would have paid an estimated $2.1 billion in discounts under the Medicare Discount Program. Panel B excludes 5 outlier drugs with coverage gap rebates exceeding 30% of gross spending; these drugs collectively accounted for $550 000 in gross Medicare Part D Spending in 2022.

In aggregate, MDP discounts were more equally distributed across drugs in proportion to their Medicare spending (Table). In contrast, CGDs were disproportionately concentrated among drugs with many users (96.0% of discounts vs 88.5% of spending) and lower than median prices (81.0% of discounts vs 52.7% of spending).

**Table.** Gross Spending, Coverage Gap Discounts, and Estimated Medicare Discount Program Rebates in 2022^a^

Drug characteristics	Millions of $ (% of drugs)		
	Gross Medicare spending in 2022	Estimated Coverage Gap Discount	Estimated Medicare Discount Program rebates
All drugs, millions of $	205 467	16 821	34 530
Drugs included in subgroups^b^	187 521 (100)	15 724 (100)	31 695 (100)
No. of patients using drug, quartile			
First, fewest patients	1259 (0.7)	19 (0.1)	250 (0.8)
Second	5400 (2.9)	109 (0.7)	1068 (3.4)
Third	14 878 (7.9)	509 (3.2)	2902 (9.2)
Fourth, most patients	165 985 (88.5)	15 088 (96.0)	27 475 (86.7)
Gross Medicare spending, quartile			
First, lowest spending	119 (0.1)	10 (0.1)	18 (0.1)
Second	1185 (0.6)	81 (0.5)	199 (0.6)
Third	8238 (4.4)	443 (2.8)	1464 (4.6)
Fourth, highest spending	177 979 (94.9)	15 190 (96.6)	30 015 (94.7)
Mean spending per patient (ie, price), quartile^c^			
First, lowest prices	9189 (4.9)	654 (4.2)	1090 (3.4)
Second	89 678 (47.8)	12 081 (76.8)	13 303 (42.0)
Third	27 451 (14.6)	1414 (9.0)	5087 (16.0)
Fourth, highest prices	61 203 (32.6)	1575 (10.0)	12 216 (38.5)

^a^ National spending and estimated discounts were extrapolated from the 20% random sample of Medicare claims.

^b^ Subgroups were defined at the level of individual branded products, defined based on product names in SSR Health.^3^ National drug codes in the Medicare claims data that could not be matched with a specific product in SSR Health were excluded from the subgroup analyses.

^c^ Mean spending used to calculate prices reflects point-of-care sales exclusive of any rebates negotiated by pharmacy benefit managers on behalf of Part D plans.

## Discussion

This cross-sectional study found that the replacement of the CGD program with the MDP in 2025 could result in substantially higher discounts for Medicare. Discounts would increase for most brand-name drugs but particularly for higher-priced drugs because patients taking these drugs would quickly reach the out-of-pocket spending threshold after which discounts increase from 10% to 20%.

This study has limitations; we did not account for the impact of other Inflation Reduction Act Medicare Part D design changes, including the $2000 annual out-of-pocket cap that could lead to increased use of high-cost drugs.^4^ Manufacturers may also strategically adjust prices to minimize revenue losses from the MDP. For example, because MDP discounts are based on prerebate spending, manufacturers of drugs with high rebate payments to Part D plans might lower list prices and rebates, which could affect non-Medicare payers.

## Notes

Supplement.
Data Sharing Statement
