# Supplementary material for: Medicare Part D Savings Under the Manufacturer Discount Program vs Coverage Gap Discounts
Source: JAMA Netw Open. 2025 Sep 8;8(9):e2530778. doi: 10.1001/jamanetworkopen.2025.30778 (PMC12418123; doi:10.1001/jamanetworkopen.2025.30778)
Supplement: Supplement. — Data Sharing Statement [file jamanetwopen-e2530778-s001.pdf]

Data Sharing Statement

Rome. Medicare Part D Savings Under the Manufacturer Discount Program vs Coverage Gap Discounts. *JAMA Netw Open*. Published September 08, 2025. doi:10.1001/jamanetworkopen.2025.30778

Data
Data available: No

Additional Information
Explanation for why data not available: The data from the Centers for Medicare and Medicaid Services and SSR Health are accessed under data use agreements. Others can reach out directly to access the data at their own expense.
